# Metagenomic next-generation sequencing of the microbiome dataset from the surface water sample collected from Serepok River in Yok Don National Park, Vietnam

**DOI:** 10.1016/j.dib.2022.108614

**Published:** 2022-09-17

**Authors:** Dinh Minh Tran, To Uyen Huynh, Thi Huyen Nguyen, Tu Oanh Do, Quang Vinh Nguyen, Anh Dzung Nguyen

**Affiliations:** Institute of Biotechnology and Environment, Tay Nguyen University, Buon Ma Thuot, Dak Lak 630000, Viet Nam

**Keywords:** Water microbiome, 16S rRNA metagenomic sequencing, Yok Don National Park, The dry deciduous dipterocarp forest

## Abstract

The Central Highlands region is considered as the center with the highest biodiversity in Vietnam because it has the majority of national parks such as Yok Don, Chu Yang Sin, Bidoup-Nui Ba, Ta Dung, Chu Mon Ray, and Kon Ka Kinh and nature reserves such as Ngoc Linh, Kon Chu Rang, Ea So, Nam Ka, and Nam Nung with different ecosystems [1]. Of the national parks and nature reserves, Yok Don has the most different ecosystem. Yok Don is the second biggest national park, and it is the only national park that conserves dry deciduous dipterocarp forests in Vietnam [2]. Presently, the decrease in forest area and global warming have led to the continuous reduction in microbial resources in this region. Thus, a dataset of the soil microbiome in this region has been established to explore microbial resources for conservation and further application in sustainable agricultural production in this region [3]; however, to the best of our knowledge, a dataset of water microbiome remains unknown. This work presented a microbiome dataset from surface water samples collected from Serepok River in Yok Don National Park, Vietnam. Metagenomic next-generation sequencing was used to characterize microbial communities in the sample. The raw sequence in this work was uploaded in Fastq format on NCBI, which can be accessed at https://www.ncbi.nlm.nih.gov/Traces/study/?acc=PRJNA853090. This metagenome dataset can provide valuable information on surface water microbial communities and their functionality. It can also be used for further studies on the conservation and application of indigenous microbial resources for sustainable crop production in this region.

**Specifications Table**

**Table:** 

Subject	Microbiology: Microbiome
Specific subject area	Metagenomics, Molecular biology, Bioinformatics
Type of data	Figures and Fastq files
How the data were acquired	Illumina MiSeq platform was used to conduct the 16S rRNA gene metagenomic sequencing
Data format	Raw and Analyzed
Description of data collection	Six surface water samples (approximately 300 mL each) were collected from six positions (0–30 cm in depth) of Serepok River in Yok Don National Park in the dry season (on January 07, 2022), and combined into one representative sample. Bacterial metagenomic DNA from the representative sample was extracted using the DNeasy PowerSoil kit (Qiagen, Germany), and V1–V9 regions of the 16S rRNA genes were amplified. Libraries of 16S rRNA gene amplicons were prepared using the Swift amplicon 16S plus ITS panel kit (Swift Biosciences, USA). Finally, Illumina MiSeq platform (2 × 150 bp paired ends) was used to sequence the prepared library [3,4].
Data source location	• Institution: Yok Don National Park • District/Province/Region: Buon Don, Dak Lak, the Central Highlands • Country: Vietnam • Latitude and longitude coordinates for collected samples: 12°55′42′′N–107°43′31′′E, 12°55′17′′N–107°46′12′′E, 12°54′31′′N–107°45′47′′E, 12°54′30′′N–107°45′12′′E, 12°54′49′′N–107°44′38′′E, 12°54′14′′N–107°43′31′′E
Data accessibility	Repository name: the National Center for Biotechnology Information (NCBI) Data identification number: PRJNA853090 Direct URL to data: https://www.ncbi.nlm.nih.gov/Traces/study/?acc=PRJNA853090

**Value of the Data**

• The data provide basic information on the microbial community of the surface water sample collected from Serepok River in Yok Don National Park, the Central Highlands, Vietnam, and its functionality.
• The data could be used to compare water microbiome profiles obtained from Yok Don National Park with those obtained from other parks in Vietnam.
• The data could be used for further studies on the conservation and application of indigenous microbial resources for sustainable crop production in the region and other related fields.

## 1 Data Description

### 1.1 Taxonomic Analysis of Water Microbiome in the Sample

**Fig. 1:**
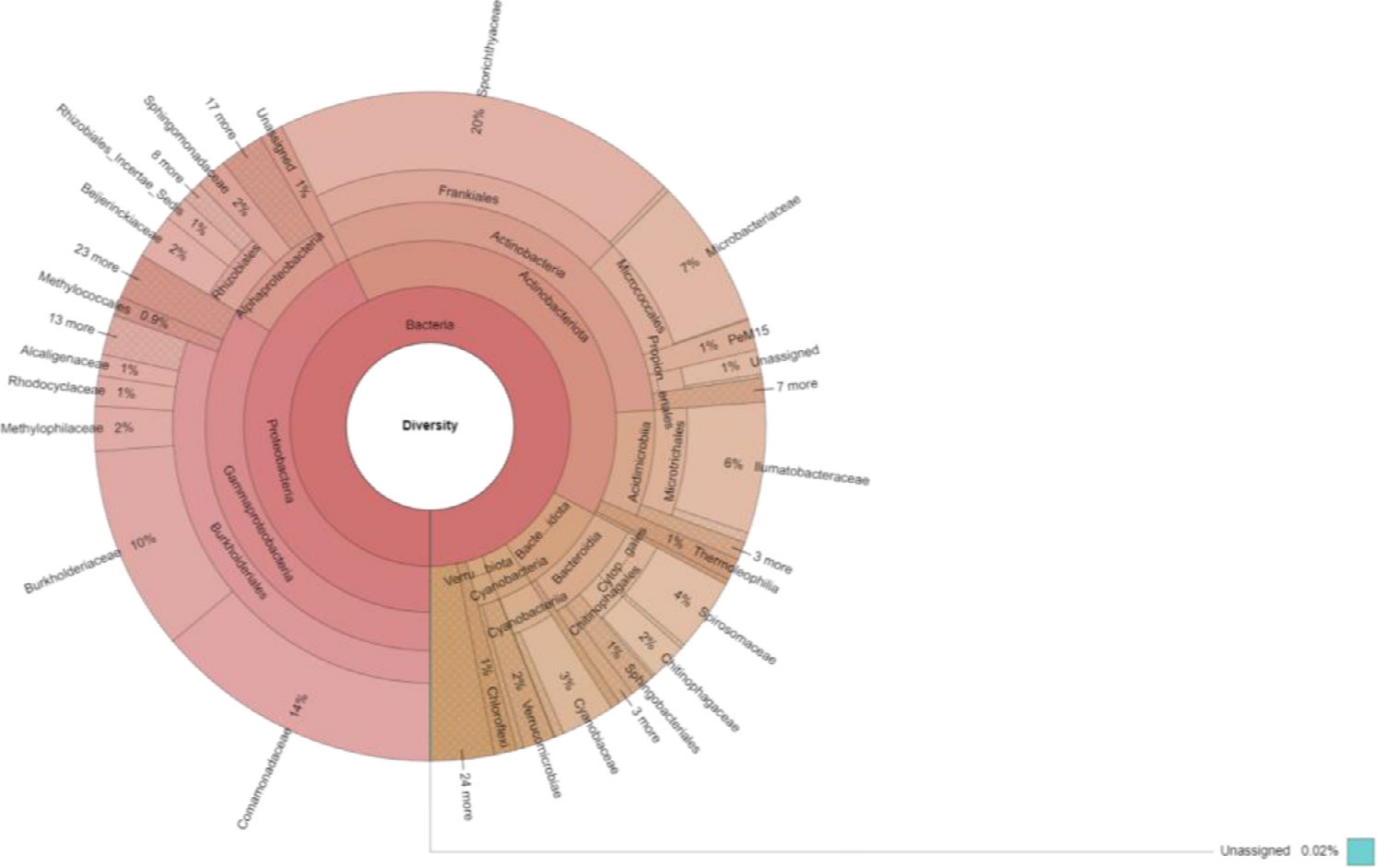
Taxonomic profiles of the water microbiome from Yok Don National Park in the Central Highlands region, Vietnam.

In this work, 290,243 out of 290,291 reads were identified. Twenty-nine phyla were detected, of which Proteobacteria (42.7%) were the most popular, followed by Actinobacteriota (40.12%) and Bacteroidota (8.1%). Sixty-seven classes were detected from the sample, of which Gammaproteobacteria (33.55%) were found to be the primary class, followed by Actinobacteria (31.14%), Alphaproteobacteria (8.03%), Acidimicrobiia (7.66%), and Bacteroidia (7.63%). Of the 158 orders identified, Burkholderiales (30.4%) were the most abundant, followed by Frankiales (19.88%), Micrococcales (7.27%), Microtrichaless (6.81%), and Rhizobiales (4.37%). Moreover, we identified 231 families, of which Sporichthyaceae (19.61%), Comamonadaceae (13.92%), Burkholderiaceae (9.89%), Microbacteriaceae (7.19%), and Ilumatobacteraceae (6.32%) were shown to be popular compared with the other identified families. Finally, 302 (61.5%) out of 491 genera were identified (Fig. 1).

**Fig. 2:**
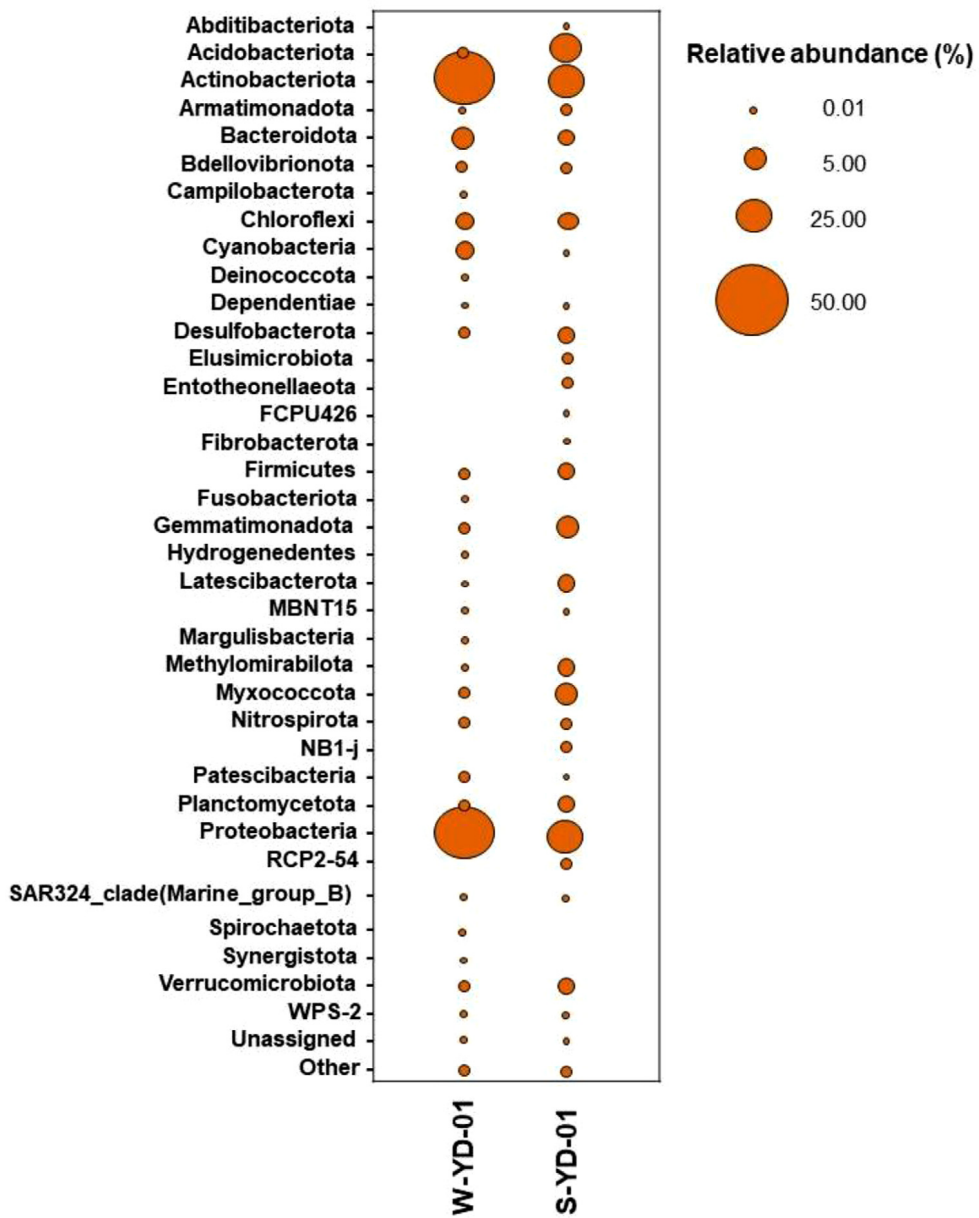
Comparision of bacterial communities in the water and soil sample collected from Yok Don National Park in the Central Highlands region, Vietnam.

Note: W-YD-01, data from this work; S-YD-01, data from Tran et al. [3]

A comparison of taxonomic profiles of microbial communities in the water sample (this study) and soil sample collected from Yok Don [3] revealed that the microbiome in the soil was more abundant than that in water. However, the phyla Proteobacteria and Actinobacteriota were found more frequently in water than in soi. Moreover, many phyla were detected in the soil sample but not in water, such as Abditibacteriota, Elusimicrobiota, Entotheonellaeota, and Fibrobacterota (Fig. 2).

### 1.2 Functional Analysis of Water Microbiome in the Sample

**Fig. 3:**
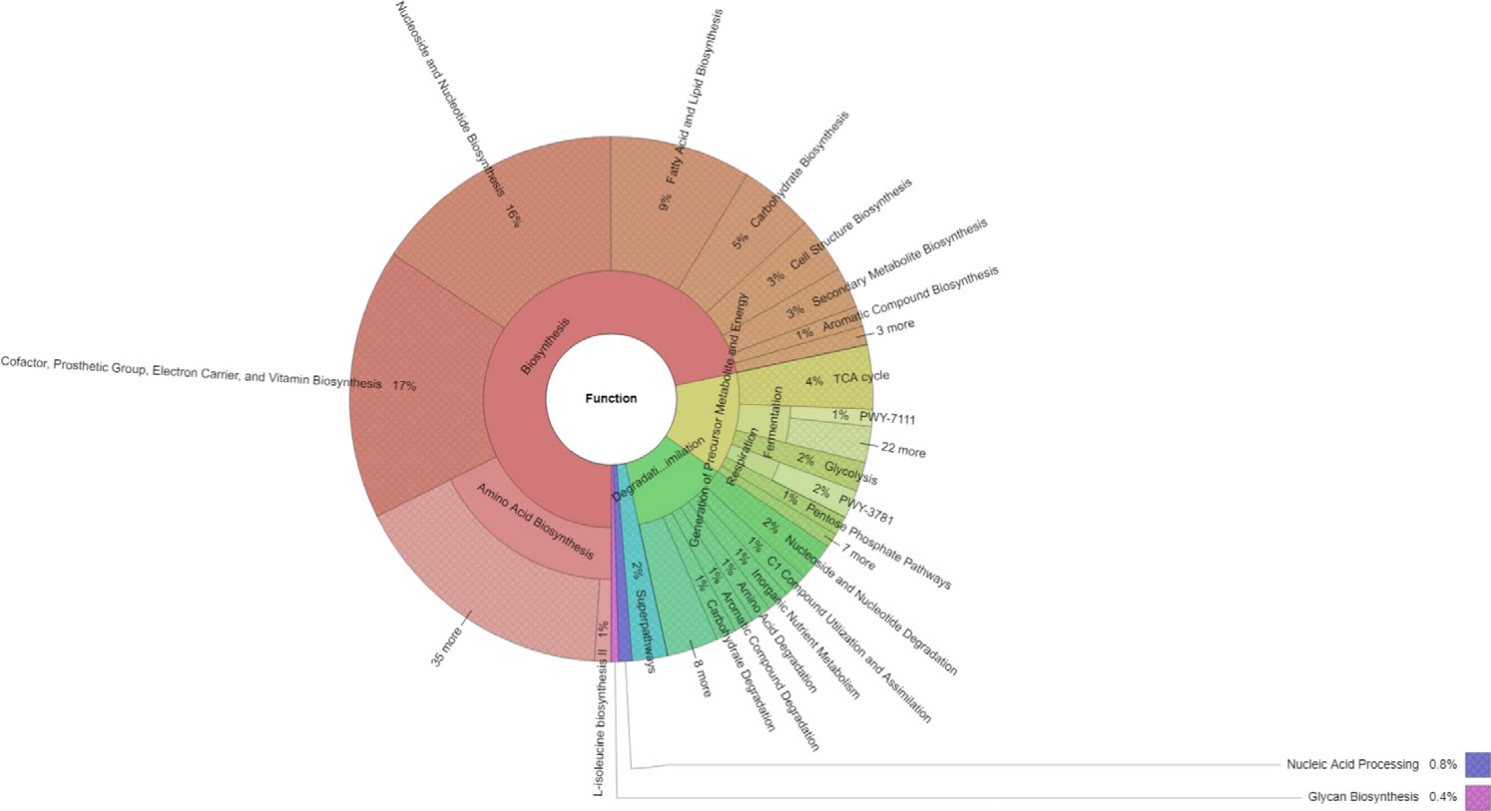
Functional profiles of the water microbiome from Yok Don National Park in the Central Highlands region, Vietnam.

Functional analysis based on the metagenomic sequence showed that functionality involved biosynthesis (71.66%), which was the primary metagenomic function of the microbial community in the water sample, followed by the generation of precursor metabolite and energy (12.82%) and the degradation/utilization/assimilation of inorganic nutrient metabolism (12.08%). Among the functions involved in biosynthesis, amino acid biosynthesis (18.11%) was the most predominant, followed by cofactor, prosthetic group, electron carrier, and vitamin biosynthesis (16.66%); nucleoside and nucleotide biosynthesis (15.64%); fatty acid and lipid biosynthesis (8.72%); carbohydrate biosynthesis (4.6%); cell structure biosynthesis (3.46%); and secondary metabolite biosynthesis (2.51%) (Fig. 3).

## 2 Experimental Design, Materials and Methods

### 2.1 Water Sample Collection

Six surface water samples (approximately 300 mL each) were collected from six positions (0–30 cm in depth) of Serepok River in Yok Don National Park in the dry season (on January 07, 2022). Sampling was performed in triplicate. Next, water samples were combined into one representative sample. Finally, the representative sample was stored at 4 °C, transferred to the laboratory, and kept at −80 °C until bacterial metagenomic DNA extraction [3,4].

### 2.2 Bacterial Metagenomic DNA Extraction, Library Preparation, and Metagenomic Sequencing

Three hundred microliters of the sample was used to extract bacterial metagenomic DNA using the DNeasy PowerSoil kit (Qiagen, Germany). Library preparation and metagenomic sequencing were performed as previously described. In brief, V1–V9 regions of the 16S rRNA gene of bacteria obtained from the sample were amplified. Then, libraries of 16S rRNA gene amplicons were prepared using the Swift amplicon 16S plus internal transcribed spacer panel kit (Swift Biosciences, USA). Finally, the Illumina MiSeq platform (2 × 150 bp paired ends) was used to sequence the 16S rRNA gene amplicon from the library [3,4].

### 2.3 Taxonomic and Functional Analyses

Taxonomic and functional profiles of bacteria in the sample were analyzed as previously described [3,4]. In brief, raw base call files were demultiplexed using bcl2fastq. Adapters, primers, and low-quality sequences (average score of <20 and read length of <100 bp) were removed using Cutadapt version 2.10 and Trimmomatic version 0.39. Furthermore, reads were clustered and dereplicated into amplicon sequence variants using QIIME2 pipeline version 2020.8 and q2-dada2 plugin. Taxonomic profiles of the water microbiome were analyzed using QIIME2 aligned with the SILVA SSURef reference database. Finally, functional profiles of the water microbiome were predicted using PICRUSt2 version 2.3.0-b and MetaCyc databases.

## Ethics Statements

None.

## CRediT authorship contribution statement

**Dinh Minh Tran:** Conceptualization, Methodology, Resources, Investigation, Formal analysis, Software, Data curation, Validation, Visualization, Writing – original draft, Writing – review & editing. **To Uyen Huynh:** Investigation, Formal analysis, Resources. **Thi Huyen Nguyen:** Investigation, Formal analysis. **Tu Oanh Do:** Investigation, Formal analysis. **Quang Vinh Nguyen:** Investigation, Formal analysis. **Anh Dzung Nguyen:** Resources, Validation, Visualization.

## Declaration of Competing Interest

The authors declare that they have no known competing financial interests or personal relationships that could have appeared to influence the work reported in this paper.

## Data Availability

Water microbiome dataset from Yok Don National Park in the Central Highlands region, Vietnam, analyzed by metagenomic next-generation sequencing (Original data) (Water microbiome)
